# Modeling biochemical pathways in the gene ontology

**DOI:** 10.1093/database/baw126

**Published:** 2016-09-01

**Authors:** David P. Hill, Peter D’Eustachio, Tanya Z. Berardini, Christopher J. Mungall, Nikolai Renedo, Judith A. Blake

**Affiliations:** 1 The Jackson Laboratory, Bar Harbor, ME 04609, USA; 2 Department of Biochemistry and Molecular Pharmacology, NYU School of Medicine, New York, NY 10016, USA; 3 Arabidopsis Information Resource, Phoenix Bioinformatics, Redwood City, CA 94063, USA; 4 Genomics Division, Lawrence Berkeley National Laboratory, Berkeley, CA 94720, USA

## Abstract

The concept of a biological pathway, an ordered sequence of molecular transformations, is used to collect and represent molecular knowledge for a broad span of organismal biology. Representations of biomedical pathways typically are rich but idiosyncratic presentations of organized knowledge about individual pathways. Meanwhile, biomedical ontologies and associated annotation files are powerful tools that organize molecular information in a logically rigorous form to support computational analysis. The Gene Ontology (GO), representing Molecular Functions, Biological Processes and Cellular Components, incorporates many aspects of biological pathways within its ontological representations. Here we present a methodology for extending and refining the classes in the GO for more comprehensive, consistent and integrated representation of pathways, leveraging knowledge embedded in current pathway representations such as those in the Reactome Knowledgebase and MetaCyc. With carbohydrate metabolic pathways as a use case, we discuss how our representation supports the integration of variant pathway classes into a unified ontological structure that can be used for data comparison and analysis.

## Introduction

Diverse biological pathways are represented as ordered molecular transformations. Biological pathway resources collect deep contextual information about pathways. For example, the Reactome Knowledgebase (1, 2), captures molecular details of human processes including signal transduction, transport, DNA replication, protein synthesis, and intermediary metabolism. In Reactome, these processes have been deconstructed to generate an ordered network of molecular transformations resulting in an extended version of a classic map of intermediary metabolism (http://www.roche.com/sustainability/for_communities_and_environment/philanthropy/science_education/pathways.htm) in which transport, signaling and association/dissociation processes are represented like the chemical transformations of classic metabolism. Pathways are routes to connect molecules of interest within the map. In some cases only single routes between molecules are possible; in others multiple routes exist, and usage of alternative routes may vary between organisms, and within an organism by tissue, cellular location and physiological state. In those more complex cases, pathway resources can create distinct pathways to represent alternate routes or locations. For example, the initial steps in the conversion of cholesterol to bile salts differ according to the tissue in which the cholesterol is located and the Reactome Knowledgebase represents these three variant routes as multiple pathways, via 7-alpha-hydroxycholesterol, via 24-hydroxycholesterol, and via 27-hydroxycholesterol (http://www.reactome.org/PathwayBrowser/#R-HSA-192105.1).

This multiplicity of pathway annotations, however, can impede integration and analysis of data across resources. Biomedical ontologies can be considered graph-based representations of the relationships among biological classes or types. They provide tools that address the multiplicity problem, first by supplying rigorous, unambiguous descriptions of these classes and the relations between them and by integrating those descriptions in a class hierarchy. The Gene Ontology (GO) provides terms and relationships to describe the Molecular Functions of gene products, their roles in Biological Processes, and their organization into Cellular Components (3–5). These root GO classes fit into the larger Basic Formal Ontology (6). In that context, Molecular Functions and Biological Processes respectively represent fine- and coarse-grained occurrents, that is, they are processes that unfold over time. Cellular Components are continuants, that is, they are entities that continue to exist over time. From a biological pathway viewpoint, an occurrent is the act of changing, and the continuant is the entity that changes (6, 7). ‘Because of their formal rigor’, ontologies enable development of computational tools that can integrate and analyze the diverse sets of data associated with ontology classes and stored in independent databases. Reasoning tools can use the classes and their computable definitions based on relationships to other terms to identify missing or logically inconsistent relationship assertions and suggest plausible attributes for entities that have not been experimentally studied.

Here, we describe a method to extend and refine the GO classes to provide a more comprehensive, consistent and integrated representation of pathways within the ontology. With the use case of carbohydrate metabolic pathways from Reactome and MetaCyc (8), we have tested the ability of this representation to support the integration of variant pathways into a unified view that can be used for data comparison and analysis.

## Results

To bring pathways into the realm of GO, we start with root terms of the ontology. A GO Biological Process (**GO:0008150**) [root term] is ‘any process specifically pertinent to the functioning of integrated living units: cells, tissues, organs, and organisms. A process is a collection of molecular events with a defined beginning and end’. Based on this definition, a biochemical pathway can be represented as a GO Biological Process. A GO Molecular Function (**GO:0003674**) [root term], in turn, represents an ‘elemental activity, such as catalysis or binding, describing the actions of a gene product at the molecular level’. In the representation of a biochemical pathway, a GO Molecular Function represents an activity that mediates a single molecular transformation (e.g. chemical transformation, transport, binding) that takes place within the pathway.

### Relationships are used to define and distinguish biological processes

Relationships can be, and to some extent have been, created between GO Molecular Function classes and GO Biological Process classes. These relationships identify functions associated with particular biochemical pathways (4). In the context of pathway representations, when particular Molecular Functions are always executed in the context of a given Biological Process they have *part_of* relationships to that process, and Biological Processes in which all instances require the execution of an instance of a given Molecular Function have *has_part* relationships to that function (http://geneontology.org/page/ontology-relations). For example, the Molecular Function kinase activity is *part_of* the Biological Process phosphorylation because every time the activity is executed (an instance of the function) it is part of the process being executed (an instance of the process). Glycolytic Process *has_part* phosphoglycerate mutase activity: execution of this activity is required for all instances of glycolysis but phosphoglycerate mutase activity is also executed in processes that are not of the class ‘glycolytic process’, like the phosphoglycerate mutase activity needed for gluconeogenesis. If a Biological Process A *has_part* Molecular Function B, we cannot conclude that every time Molecular Function B occurs it is *part_of* Biological Process A.

**Figure 1.**
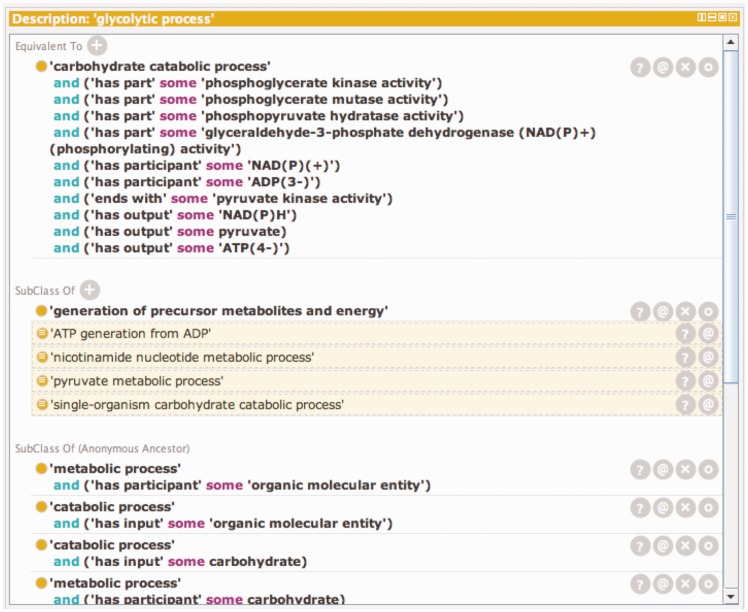
A window showing the logical definition of the class ‘glycolytic process’ using the Protégé ontology editing tool. The Protégé ontology editor (27) was used to construct, validate and display logical definitions to distinguish kinds of glycolysis. The top portion of the window shows the equivalency axioms that make up the logical definition of the term. The definition is made up of both molecular function and participant requirements. Any term in the ontology that satisfies these requirements will automatically be classified as a subtype of ‘glycolytic process’. The lower portion of the window shows the classes of which ‘glycolytic process’ is a subtype. The non-highlighted class is an asserted statement and the highlighted classes are inferred based on the logical definition of ‘glycolytic process’ and the logical definition of those classes.

Temporal order is not usually specified in GO Biological Processes, but two subproperties of the *has_part* relation defined in the OBO Relations Ontology, *starts_ with* (**RO_0002224**) and *ends_with* (**RO_0002230**) (with the persistent URL http://purl.obolibrary.org/obo/BFO_0000051), can be used to define the boundaries of a Biological Process. Relationships like these can also be used to define process classes in GO by creating equivalence statements or logical definitions which when combined are necessary and sufficient to represent the class. Logical definitions can also be used to infer class inclusion because they satisfy necessary and sufficient conditions for another class. The logical definition and inferred classes for the GO term ‘Glycolytic Process’ are shown in Figure 1.

### GO representations of biochemical pathways are species- and state-neutral

Representations of general biochemical pathways created with GO Biological Process and Molecular Function terms are neutral with respect to phylogenetic origin or physiological state (3). This neutrality in representing biochemical pathways presents a challenge because different species, different tissues within one species, or a tissue under different physiological conditions, may use different enzymatic activities or substrates to carry out processes that achieve the same biochemical end, as in the case of the differentially expressed and regulated isoforms of several of the enzymes that mediate steps of glycolysis in humans and mice (9). The representation of a general pathway class in GO thus needs to account for the totality of these variations. It must support annotation of all instances of that process and distinguish the use of different gene products under different physiological conditions while simultaneously collating conserved Molecular Functions over large phylogenetic distances.

### Carbohydrate metabolic pathways provide a use case to demonstrate representations of biological pathway classes in a GO context

**Figure 2.**
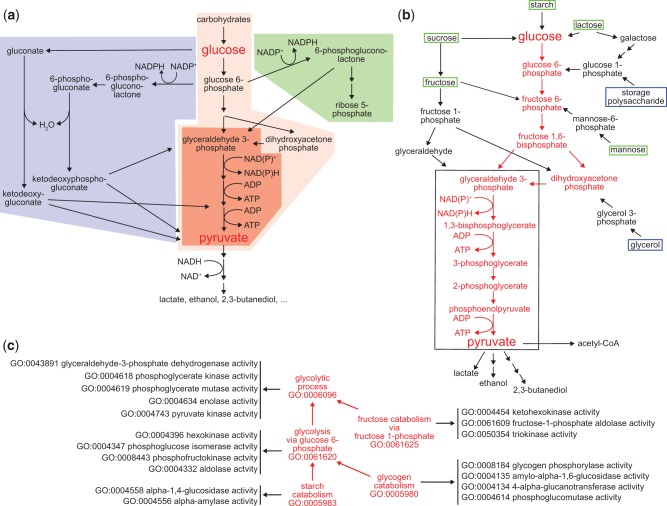
Pathways for carbohydrate catabolism. **(a)** Three evolutionarily old and interrelated processes, the Entner-Doudoroff pathway (blue), glycolysis (red) and the pentose phosphate pathway (blue) convert carbohydrates to pyruvate, yielding both energy as ATP and reducing equivalents, and pyruvate and other small molecules that can be further catabolized or consumed as biosynthetic intermediates. The very well conserved core pathway of glycolysis is highlighted with intense red shading. (b) The molecules and reactions that make up canonical glycolysis, the conversion of glucose to pyruvate, are shown in red. Reactions found in diverse taxa that bring other carbohydrates into this process are shown in black, as are three examples of fermentation of pyruvate and its oxidative decarboxylation to acetyl-CoA, at the bottom of the diagram. Boxes distinguish carbohydrate substrates that a mammal typically recovers from the environment (green) from ones synthesized internally (blue). The black outline identifies the core group of molecules and reactions shared by all glycolytic pathways, which differentiate them from other metabolic processes. **(c)** The kinds and components of glycolytic catabolism can be represented as an ontology. Glycolytic pathways (processes) are related to a core glycolytic process by *is_a* relationships (red arrows). Each form of glycolytic process has as parts (black arrows) specific Molecular Functions that define it and distinguish it from other forms of glycolysis. Core glycolysis and four other terms are shown; a complete set of process terms with the *is_a* relationships for each is given in Supplementary Table S1.

Here we use glycolysis as a test case for applying GO pathway representations to specific biochemical processes. Glycolysis is the evolutionarily ancient group of processes that convert glucose and other carbohydrates to pyruvate while reducing NAD(P)^+ ^ to NAD(P)H and converting ADP to ATP (10–13). In present-day organisms glycolysis is closely intertwined with related processes of carbohydrate metabolism such as the Entner-Doudoroff pathway and the pentose phosphate shunt (Figure 2a), as well as the possibly even more ancient process of gluconeogenesis (14, 15). Under anaerobic conditions, continued glycolysis requires a fermentation process in which the NAD(P)^+ ^consumed in the conversion of glyceraldehyde-3-phosphate to 1,3-bisphosphoglycerate is regenerated by additional chemical reactions that reduce pyruvate to lactate, ethanol, 2,3-butanediol or other molecules (11).

Therefore, the glycolytic process as a class represents a variety of different processes—all of which can be considered subclasses of glycolysis. Even within a single organism many routes may lead to the formation of glucose-6-phosphate from carbohydrate starting materials (Figure 2b). One major route leads from glucose-6-phosphate to the formation of dihydroxyacetone phosphate and glyceraldehyde-3-phosphate. Several carbohydrate starting materials yield molecules that feed into this route at intermediate steps. Only one route, however, mediates the conversion of glyceraldehyde-3-phosphate to pyruvate and this is conserved in all subclasses of glycolysis. Variant glycolytic pathways mediate the conversion of other carbohydrates into intermediates in the core pathway and fermentative pathways couple the further metabolism of pyruvate to regeneration of NAD+ under anaerobic conditions.

### GO Biological Process definitions identify, classify, and distinguish each of the variant glycolytic processes

**Figure 3.**
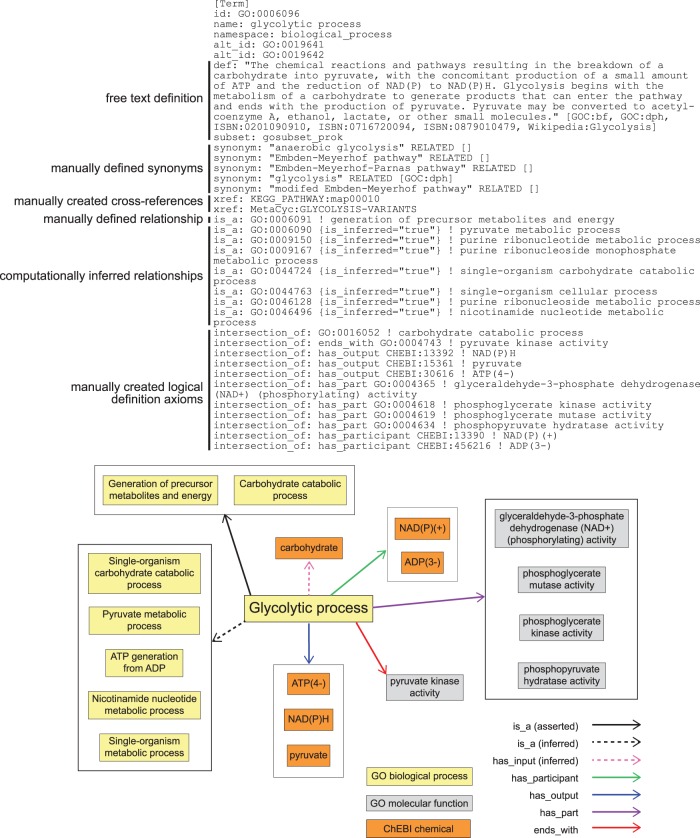
GO definition of ‘glycolytic process’ (GO:0006096) in OBO format. Key elements include a free text definition and synonyms, manually composed by a human expert curator, manually identified cross-references to representations of this process in other databases, a manually identified relationship to a parental GO Biological Process term, computationally inferred relationships to parental GO Biological Process terms (*is_inferred*=‘true’), and manually identified assertions that collectively are sufficient to identify this process and distinguish it from all other Biological Processes in GO (*intersection_of*). The lower panel graphically represents the elements of the definition and their relationships to one another.

To represent these pathways and the relationships among them within the logical structure of GO, we identified the conserved aspects of all glycolytic pathways and used those to create a logical definition based on necessity and sufficiency for the most general class, ‘glycolytic process’ (16). We next represented variations that had aspects not universally conserved as subclasses, e.g. glycolysis via glucose 6-phosphate (Table 1, Figure 2c). Each subclass of glycolytic process is defined by assertions that restrict the parent (superclass) process definition by specifying additional functions and participants. The GO Molecular Functions or sub-processes that distinguish the generic process from other variants are necessary parts (*has_ part*, *starts_with*, *ends_with*), as are key input, intermediate, and output chemical entities (*has_i nput* (**RO_0002233**), *has_participant* (**RO_0000057**), *has_output* (**RO_0002234**), from the OBO Relations Ontology with the persistent URL http://purl.obolibrary.org/obo/RO_0000057) (Figure 3). These chemical entities are taken from the ChEBI (Chemical Entities of Biological Interest) ontology (17). These definitions do not describe a particular kind of glycolytic process fully but rather identify a minimum set of features sufficient to distinguish and uniquely identify it. Thus, ‘glycolytic process’ (**GO:0006096**), the broadest term used to describe glycolysis, is a subclass of ‘carbohydrate catabolic process’, and is defined by features that include NAD(P)H and ATP outputs and the participation of the enzymatic activities that convert glyceraldehyde 3-phosphate to pyruvate (Figure 3). Its children include ‘glycolytic process from glycerol’ (**GO:0061613**), distinguished by several features including that glycerol is an input (Figure 2b). This approach to describing variant processes allows easy identification of subprocesses shared among a variety of different pathways, thereby clarifying the relationships among the pathways.

**Table 1.** Kinds of glycolysis

Start	Entry point to glycolysis	GO term
Core	dihydroxyacetone phosphate (DHAP), glyceraldehyde 3-phosphate (G3P)	**GO:0006096**
Canonical	glucose → glucose 6-phosphate → fructose 6-phosphate → fructose 1,6-bisphosphate → DHAP,G3P (core)	**GO: 0061621**
Glycerol	glycerol 3-phosphate → DHAP (core)	**GO: 0061613**
Fructose	fructose 1-phosphate → DHAP, G3P (core)	**GO: 0061625**
Galactose	glucose 1-phosphate → glucose 6-phosphate (canonical)	**GO:0061623**
Mannose	mannose 6-phosphate → fructose 6-phosphate (canonical)	**GO:0061619**
Storage polysaccharide	glucose 1-phosphate → fructose 6-phosphate (canonical)	**GO:0093001**

### Glycolytic fermentations couple glycolysis to NAD+ regeneration

Glycolysis converts NAD(P)^+ ^to NAD(P)H. To proceed under anaerobic conditions, it must be coupled to a fermentation process that supports NAD(P)^+ ^regeneration. To test whether our method of modeling glycolytic pathways can be extended to related domains of metabolism, we have extended our methodology to include aspects of fermentation. Glycolytic fermentation and its children describe these processes and necessarily start with some kind of glycolytic process. The more specific process ‘glycolytic fermentation to ethanol’ (**GO:0019655**) e.g. is a subclass (*is_a* child) of ‘glycolytic fermentation’ that starts with the glycolytic process ‘glycolytic process through fructose-6-phosphate’, has glucose as an input, and ethanol as an output. The relationship of the fermentation process and glycolysis is shown as a metabolic pathway in Figure 2b; the definitions of the GO fermentation terms are in supplement Table 1.

### Organizing the diverse classes of glycolysis and glycolytic fermentation through a process of subsumption clarifies and tests the functional and logical links among these processes and between them and other forms of carbohydrate catabolism

Modular representation of variant processes is not an approach unique to GO. Pathway databases such as MetaCyc construct modules to group metabolic processes such as variant forms of glycolysis or pathways for catabolism of an amino acid (8). The structure of GO, however, further enables us to use the definition elements of related processes to specify the nature of these relationships explicitly and consistently in a process of subsumption. We named conserved processes based on shared intermediate substances, such as glucose-6-phosphate, but the central focus of GO is occurrents, the Biological Processes and Molecular Functions, that are enabled by gene products. This strategy of successively grouping process occurrents based on shared subprocess occurrents allows creation of a hierarchy that nests glycolytic processes in a useful way: ‘glycolytic process through glucose-6-phosphate’ is a subclass of ‘glycolytic process through fructose-6-phosphate’ which in turn is a subclass of ‘glycolytic process’ (Figure 1b and c and Supplementary Table S1). Each subclass has all of the characteristics of all of its parents and is further differentiated from its superclasses by assertions that distinguish it from them and other subclasses. These assertions are represented in equivalence axioms in Protégé. For example the process ‘canonical glycolysis’ is the specific kind of glycolytic pathway that converts glucose to pyruvate and is modeled as a subclass of ‘glycolysis through glucose-6-phosphate’ with an input of glucose and a necessary first step of glucokinase activity. ‘Canonical glycolysis’ also necessarily includes all of the Molecular Functions required for the execution of its superclasses and includes the output of pyruvate from the generic superclass ‘glycolytic process’.

### Subsumed process definitions support powerful logical reasoning tools

Creating equivalent-class definitions for pathway classes allows use of an automated reasoning tool, ELK, to infer additional classifications. For example, since ‘glycolytic process’ is a ‘catabolic process’ with input carbohydrate and ‘canonical glycolysis’ is a subclass of ‘glycolytic process’ with input glucose, ‘canonical glycolysis’ is automatically classified as a subclass of ‘glucose catabolic process’ (Figure 2). Supplementary Table S1 shows inferred relationships (lines with the label *is_inferred *=* *true) for all glycolysis process terms.

This reasoning extends to the chemical entities involved in a process as well. Thus, glycolysis *is_a* purine ribonucleoside metabolic process (**GO:0046128**) and *is_a* nicotinamide nucleotide metabolic process (**GO:0046496**). These inferences are correct because ADP and ATP and NAD(P)^+ ^and NAD(P)H are interconverted (metabolized) in the course of glycolysis. These relationships may not be part of the textbook description of glycolysis but without them, glycolysis cannot proceed.

Conversely, ‘sulfoglycolysis’, the catabolism of sulfoquinovose (glucose-6-sulfonate) to pyruvate and 2,3-dihydroxypropane-1-sulfonate (18) is not a subclass of glycolysis because its input molecule is not a carbohydrate (a molecule that satisfies the formula C_*m*_(H_2_O)_*n*_) but rather a carbohydrate derivative. This is a well-established chemical distinction. By respecting this distinction in our process definitions, we maintain consistency between GO and ChEBI (19). More importantly, we maintain a separation between two processes, glycolysis and sulfoglycolysis, with distinct evolutionary histories and biological roles (18), and a structure of logical relationships in GO that is consistent and easily parsed and modified if chemical definitions were changed.

## Discussion

Our work here shows a strategy for representing biochemical pathways in GO by creating logical definitions (equivalent class expressions) for Biological Processes that specify both the Molecular Functions executed in those pathways and the chemical entities that participate in the pathways. These logical definitions provide computationally tractable necessary and sufficient conditions to automatically classify metabolic processes in GO. The approach of creating logical definitions has been used successfully to define classes in GO and in other OBO ontologies (20–23).

We have tested our representation of glycolytic processes in GO by manually exploring the alignment to two pathways databases, Reactome and MetaCyc. We found that our representation of process could be cross-referenced to these resources and that when these resources represented pathways that were not originally accounted for in our model, we could easily extend our model to include them or to exclude them based on the constraints of our logical definitions (Figure 3 and Supplementary Table S1). The annotation and analysis strategies described here are applicable to any aspect of cellular biology that can be expressed as reactions organized into pathways. Using our strategy, we have successfully modeled other metabolic pathways in GO including the aerobic and anaerobic fates of pyruvate, sulfoglycolysis and molybdopterin metabolism.

### Can this mapping strategy be generalized further?

Our work has focused on very well-studied metabolic processes. Glycolysis presented a particularly challenging use case because, being critical for primary metabolism, it has evolved to utilize a variety of substrates and its enzymes have been coopted in other metabolic pathways. Therefore, creating necessary and sufficient definitions for the generic glycolytic process and its subtypes involved continuous refinement. We are confident that the use case we have developed is a model for mapping a very broad range of biological processes. Work on the Reactome project has already established that the molecular details of complex aspects of developmental biology, cell cycle progression and immune function can be captured in a reaction—pathway data model (1, 2), so mapping these processes in GO using the strategy described here should require no new concepts or tools.

Our work shows that the modular approach of creating common processes with necessary Molecular Functions and grouped by common intermediates is both rigorous and extensible. By rigorous, we mean that collections of functions can be grouped to create modules that describe conserved parts of biochemical pathways and that, together with common participants, categorize the pathways in a logically consistent and biologically meaningful way. By extensible, we mean that those modules can be used to describe variants of the pathway. This concept was proven in principle in both our own work and in our effort to map our representation onto external resources. In an example of our own work, we first described the generic glycolytic process module that represented the input of a carbohydrate and the output of pyruvate combined with the last five enzymes common to all glycolytic pathways. We then further extended the representation by creating subclasses that accounted for the variety of molecules that can feed into the pathway such as ‘glycolysis from glycerol’ and ‘glycolysis from mannose’.

The structural integration described here for GO, Reactome, and MetaCyc provides a basis for extensions of GO definitions to include directionality of reactions and systematic annotation of input and output entities. These extensions will facilitate accurate identification of equivalent pathways in databases, e.g. between Reactome and MouseCyc (24). Although the structural organization is consistent between pathway databases and GO, the focus of the resources is different. Representations of pathways in MetaCyc and Reactome focus on transformations of chemical entities, while the representation in GO remains focused on the processes that are encoded by gene products. This structural integration is also a general model for imposing a rigorous logical structure on large sets of biomedical data to support seamless data mining, integration, and computational reasoning across multiple resources, and for potential future automation of major parts of the process of ontology construction and data tagging.

### Can the mapping process described here be scaled up?

Our work involved one group of biological processes and was carried out manually by expert curators and software developers. Two challenges to address in future work are to develop strategies to streamline and at least partially automate the mapping process to handle the very large amounts of information available in ontologies and pathway resources, and to explore crowdsourcing strategies that might enable people who are not domain experts to participate effectively in these ontology development and mapping projects.

By using the OBO Foundry standard for interoperable ontologies (25), it becomes possible to integrate data on cell components, cell types, anatomical structures and phenotypes into this annotation and data mining process and to extend it from the chemical reactions of intermediary metabolism to the broader array of molecular transformations contained in pathway databases like Reactome. By enabling examination of logical consistency in pathway representations, such as the inference of glycolytic processes initiating with different kinds of carbohydrates and their classification as types of that kind of carbohydrate catabolism, this approach provides the ability to look at specific dependencies and interactions between pathways, metabolites, and cellular processes.

## Experimental procedures

The term definitions that make up GO are freely available in both OBO and OWL data formats from the GO web site (http://geneontology.org/page/download-ontology). The work described here used the core editor’s version of GO, gene_ontology_write.obo, and imported external ontologies. Ontologies can be converted between OBO and OWL formats using owltools (https://github.com/owlcollab/owltools). The OBO format was used for human readability during the manual quality checking and for work in OBO-Edit and the OWL format was used for work in Protégé. The core editor’s version of GO is uploaded to the GO repository by conversion to obo format where it is processed into downstream files that are available for User consumption. Ontology editing was performed according to the standard procedures of The Gene Ontology Consortium (http://geneontology.org/page/go-editor-guides). The OBO-Edit ontology editing tool was used to add new terms to the ontology and to view and edit the structure of the ontology in graphical formats (26). Protégé (27) was used to create logical (equivalent class) definitions of processes and to visualize inferred relationships. The construction of logical definitions (equivalences) was done manually, using standard textbooks for the initial identification of Molecular Functions and key chemicals that are used to carry out Biological Processes. These initial logical definitions were further refined by thorough searching of the biomedical literature and by manual examination of the existing representation of biochemical pathways in the Reactome and MetaCyc databases (8).

Within Protégé we used the ELK reasoner plugin for Protégé to identify inferred relationships between new and modified terms, following previously validated protocols (28, 29). Once inferences were identified, an iterative process was undertaken in which we manually checked them for accuracy and correctness based on published literature and our knowledge. If errors were spotted, the underlying assertions in logical definitions that contributed to the errors were modified by addition of further restrictions, or removal or modification of specified relations. The most common errors were incorrect inference due to insufficient specificity of logical definitions as new classes were added or too restrictive specificity as more pathways of a given class were identified. As an example of the first case, if we initially defined ‘glycolytic process’ as a catabolic process that has an input of a carbohydrate and an output of pyruvate and we defined the Entner-Doudoroff pathway as a ‘glucose catabolic process’ that has an output of pyruvate, then the Entner-Doudoroff pathway would be misclassified as a type of ‘glycolytic process’. To correct this error we would need to modify the definition of ‘glycolytic process’ by increasing its specificity so that the definition of the Entner-Doudoroff pathway no longer satisfies it. As an example of the second case, if we had defined ‘glycolytic process’ as a catabolic process that has an input of glucose and an output of pyruvate, then glycolysis from sucrose would not be correctly classified as subtypes of glycolytic processes because sucrose is not a type of glucose. In order to achieve the correct classification, we need to relax the definition of glycolytic process to include compounds other than glucose, but keep it stringent enough not to include compounds whose catabolism would not be considered a type of glycolytic process. Stringencies of definitions were checked with each addition of a new class identified from Reactome, Metacyc or the published literature. Stringencies of definitions were increased by the addition of necessary molecular functions or participants to a process or my making the participants more specific. Stringencies of definitions were decreased by removing molecular functions that were deemed unnecessary, by removing participants, or by making participants more general.

## Supplementary data

Supplementary data are available at *Database* Online.

Supplementary Data
