# Supplementary material for: Modeling biochemical pathways in the gene ontology
Source: Database (Oxford). 2016 Sep 1;2016:baw126. doi: 10.1093/database/baw126 (PMC5009323; doi:10.1093/database/baw126)
Supplement: Supplementary Data [file supp_2016_baw126_index.html]

Modeling biochemical pathways in the gene ontology — Supplementary Data 

# Modeling biochemical pathways in the gene ontology

## Supplementary Data

files

- Supplementary Data - doc file
