# Supplementary material for: Modeling biochemical pathways in the gene ontology
Source: Database (Oxford). 2016 Sep 1;2016:baw126. doi: 10.1093/database/baw126 (PMC5009323; doi:10.1093/database/baw126)
Supplement: Supplementary Data [file supp_baw126_Supplemental_Table_1.doc]

**Supplemental Table 1 –set of GO terms representing glycolysis-related biological processes, in order of GO id**. Each term includes a free-text definition, synonyms, cross-references to equivalent instances in pathway databases, other GO terms that comprise the manually-annotated logical definition of the term, and computationally inferred relationships to other GO terms. Although the underlying representation of these terms is in OWL, we have shown them here in OBO format to ease human-readability.

[Term]

id: GO:0006096

name: glycolytic process

namespace: biological_process

alt_id: GO:0019641

alt_id: GO:0019642

def: "The chemical reactions and pathways resulting in the breakdown of a carbohydrate into pyruvate, with the concomitant production of a small amount of ATP and the reduction of NAD(P) to NAD(P)H. Glycolysis begins with the metabolism of a carbohydrate to generate products that can enter the pathway and ends with the production of pyruvate. Pyruvate may be converted to acetyl-coenzyme A, ethanol, lactate, or other small molecules." [GOC:bf, GOC:dph, ISBN:0201090910, ISBN:0716720094, ISBN:0879010479, Wikipedia:Glycolysis]

subset: gosubset_prok

synonym: "anaerobic glycolysis" RELATED []

synonym: "Embden-Meyerhof pathway" RELATED []

synonym: "Embden-Meyerhof-Parnas pathway" RELATED []

synonym: "glycolysis" RELATED [GOC:dph]

synonym: "modifed Embden-Meyerhof pathway" RELATED []

xref: KEGG_PATHWAY:map00010

xref: MetaCyc:GLYCOLYSIS-VARIANTS

is_a: GO:0006090 {is_inferred="true"} ! pyruvate metabolic process

is_a: GO:0006091 ! generation of precursor metabolites and energy

is_a: GO:0009150 {is_inferred="true"} ! purine ribonucleotide metabolic process

is_a: GO:0009167 {is_inferred="true"} ! purine ribonucleoside monophosphate metabolic process

is_a: GO:0044724 {is_inferred="true"} ! single-organism carbohydrate catabolic process

is_a: GO:0044763 {is_inferred="true"} ! single-organism cellular process

is_a: GO:0046128 {is_inferred="true"} ! purine ribonucleoside metabolic process

is_a: GO:0046496 {is_inferred="true"} ! nicotinamide nucleotide metabolic process

intersection_of: GO:0016052 ! carbohydrate catabolic process

intersection_of: ends_with GO:0004743 ! pyruvate kinase activity

intersection_of: has_output CHEBI:13392 ! NAD(P)H

intersection_of: has_output CHEBI:15361 ! pyruvate

intersection_of: has_output CHEBI:30616 ! ATP(4-)

intersection_of: has_part GO:0004365 ! glyceraldehyde-3-phosphate dehydrogenase (NAD+) (phosphorylating) activity

intersection_of: has_part GO:0004618 ! phosphoglycerate kinase activity

intersection_of: has_part GO:0004619 ! phosphoglycerate mutase activity

intersection_of: has_part GO:0004634 ! phosphopyruvate hydratase activity

intersection_of: has_participant CHEBI:13390 ! NAD(P)(+)

intersection_of: has_participant CHEBI:456216 ! ADP(3-)

[Term]

id: GO:0019650

name: glycolytic fermentation to butanediol

namespace: biological_process

alt_id: GO:0030646

def: "The anaerobic chemical reactions and pathways resulting in the breakdown of glucose into butanediol; effected by some members of the Enterobacteriaceae, e.g. Enterobacter, Erwinia, Klebsiella, and Serratia." [GOC:dph, GOC:nr, ISBN:0198506732]

subset: gosubset_prok

synonym: "butanediol fermentation" EXACT []

synonym: "glucose catabolic process to butanediol" EXACT [GOC:dph]

synonym: "glucose fermentation to butanediol" EXACT []

xref: MetaCyc:P125-PWY

xref: Wikipedia:Butanediol_fermentation

is_a: GO:0006007 {is_inferred="true"} ! glucose catabolic process

is_a: GO:0019364 {is_inferred="true"} ! pyridine nucleotide catabolic process

is_a: GO:0019660 ! glycolytic fermentation

is_a: GO:0034077 {is_inferred="true"} ! butanediol metabolic process

intersection_of: GO:0019660 ! glycolytic fermentation

intersection_of: ends_with GO:0000721 ! (R,R)-butanediol dehydrogenase activity

intersection_of: has_input CHEBI:17234 ! glucose

intersection_of: has_output CHEBI:62064 ! butane-2,3-diol

intersection_of: has_part GO:0003984 ! acetolactate synthase activity

intersection_of: has_part GO:0047605 ! acetolactate decarboxylase activity

intersection_of: has_part GO:0052587 ! diacetyl reductase ((R)-acetoin forming) activity

[Term]

id: GO:0019655

name: glycolytic fermentation to ethanol

namespace: biological_process

def: "The anaerobic chemical reactions and pathways resulting in the breakdown of glucose; it is converted into ethanol and carbon dioxide (CO2), producing two molecules of ATP for each molecule of glucose." [GOC:dph, GOC:nr, ISBN:0716720094]

subset: gosubset_prok

synonym: "alcoholic fermentation" RELATED [ISBN:124925502]

synonym: "ethanol fermentation" EXACT []

synonym: "glucose catabolic process to ethanol" EXACT [GOC:dph]

synonym: "glucose fermentation to ethanol" EXACT []

xref: Wikipedia:Ethanol_fermentation

is_a: GO:0006007 {is_inferred="true"} ! glucose catabolic process

is_a: GO:0006067 {is_inferred="true"} ! ethanol metabolic process

is_a: GO:0006734 {is_inferred="true"} ! NADH metabolic process

is_a: GO:0019660 ! glycolytic fermentation

is_a: GO:1902707 {is_inferred="true"} ! hexose catabolic process to ethanol

intersection_of: GO:0019660 ! glycolytic fermentation

intersection_of: has_input CHEBI:17234 ! glucose

intersection_of: has_output CHEBI:16236 ! ethanol

intersection_of: starts_with GO:0061615 ! glycolytic process through fructose-6-phosphate

relationship: has_input CHEBI:17234 {is_inferred="true"} ! glucose

relationship: has_output CHEBI:16236 {is_inferred="true"} ! ethanol

[Term]

id: GO:0019657

name: glycolytic fermentation to propionate

namespace: biological_process

def: "Glycolytic fermentation resulting in the catabolism of glucose to propionate, yielding energy in the form of ATP; an alternative to the acrylate pathway to produce propionate." [GOC:dph, GOC:nr, MetaCyc:P108-PWY]

subset: gosubset_prok

synonym: "succinate-propionate fermentation" EXACT []

xref: MetaCyc:P108-PWY

is_a: GO:0006007 {is_inferred="true"} ! glucose catabolic process

is_a: GO:0006105 ! succinate metabolic process

is_a: GO:0006734 {is_inferred="true"} ! NADH metabolic process

is_a: GO:0019541 ! propionate metabolic process

is_a: GO:0019660 ! glycolytic fermentation

is_a: GO:0042867 ! pyruvate catabolic process

intersection_of: GO:0019660 ! glycolytic fermentation

intersection_of: has_output CHEBI:17272 ! propionate

intersection_of: has_part GO:0043821 ! propionyl-CoA:succinate CoA-transferase activity

intersection_of: has_part GO:0047154 ! methylmalonyl-CoA carboxytransferase activity

intersection_of: starts_with GO:0061633 ! transport-coupled glycolytic process through glucose-6-phosphate

[Term]

id: GO:0019658

name: glucose fermentation to lactate and acetate

namespace: biological_process

def: "The anaerobic chemical reactions and pathways resulting in the breakdown of glucose to lactate and acetate, yielding energy in the form of ATP." [MetaCyc:P124-PWY]

subset: gosubset_prok

synonym: "bifidum pathway" EXACT []

synonym: "glucose catabolic process to lactate and acetate" BROAD []

xref: MetaCyc:P124-PWY

is_a: GO:0006083 {is_inferred="true"} ! acetate metabolic process

is_a: GO:0019659 {is_inferred="true"} ! glucose catabolic process to lactate

is_a: GO:0019662 ! non-glycolytic fermentation

is_a: GO:1902706 {is_inferred="true"} ! hexose catabolic process to acetate

intersection_of: GO:0009056 ! catabolic process

intersection_of: has_input CHEBI:17234 ! glucose

intersection_of: has_output CHEBI:24996 ! lactate

intersection_of: has_output CHEBI:30089 ! acetate

relationship: has_input CHEBI:17234 {is_inferred="true"} ! glucose

relationship: has_output CHEBI:24996 {is_inferred="true"} ! lactate

relationship: has_output CHEBI:30089 {is_inferred="true"} ! acetate

[Term]

id: GO:0019660

name: glycolytic fermentation

namespace: biological_process

def: "Fermentation that includes the anaerobic conversion of glucose to pyruvate via the glycolytic pathway." [GOC:curators]

subset: gosubset_prok

xref: MetaCyc:Pyruvate-Degredation

is_a: GO:0006090 {is_inferred="true"} ! pyruvate metabolic process

is_a: GO:0006113 ! fermentation

is_a: GO:0009150 {is_inferred="true"} ! purine ribonucleotide metabolic process

is_a: GO:0009167 {is_inferred="true"} ! purine ribonucleoside monophosphate metabolic process

is_a: GO:0044723 {is_inferred="true"} ! single-organism carbohydrate metabolic process

is_a: GO:0046128 {is_inferred="true"} ! purine ribonucleoside metabolic process

is_a: GO:0046496 {is_inferred="true"} ! nicotinamide nucleotide metabolic process

intersection_of: GO:0006113 ! fermentation

intersection_of: starts_with GO:0006096 ! glycolytic process

[Term]

id: GO:0019661

name: glucose catabolic process to lactate via pyruvate

namespace: biological_process

def: "The anaerobic enzymatic chemical reactions and pathways resulting in the breakdown of glucose to lactate, via canonical glycolysis, yielding energy in the form of adenosine triphosphate (ATP)." [GOC:jl]

subset: gosubset_prok

synonym: "glucose fermentation to lactate via pyruvate" EXACT []

synonym: "homofermentation" EXACT []

synonym: "homofermentative lactate fermentation" EXACT []

synonym: "homofermentative pathway" EXACT []

synonym: "homolactate fermentation" EXACT []

synonym: "homolactic fermentation" EXACT [ISBN:124925502]

xref: MetaCyc:PWY-5481

is_a: GO:0006734 {is_inferred="true"} ! NADH metabolic process

is_a: GO:0019659 ! glucose catabolic process to lactate

is_a: GO:0019660 {is_inferred="true"} ! glycolytic fermentation

intersection_of: GO:0006007 ! glucose catabolic process

intersection_of: has_input CHEBI:17234 ! glucose

intersection_of: has_intermediate CHEBI:15361 ! pyruvate

intersection_of: has_output CHEBI:24996 ! lactate

intersection_of: has_part GO:0004459 ! L-lactate dehydrogenase activity

intersection_of: starts_with GO:0061621 ! canonical glycolysis

[Term]

id: GO:0019664

name: mixed acid fermentation

namespace: biological_process

def: "The anaerobic chemical reactions and pathways resulting in the breakdown of glucose into ethanol, lactate, formate, succinate, and acetate, yielding energy in the form of ATP." [MetaCyc:FERMENTATION-PWY, PMID:124925502]

subset: gosubset_prok

synonym: "glucose catabolic process to mixed acids" BROAD []

synonym: "glucose fermentation to mixed acids" EXACT []

xref: Wikipedia:Mixed_acid_fermentation

is_a: GO:0006103 {is_inferred="true"} ! 2-oxoglutarate metabolic process

is_a: GO:0006105 {is_inferred="true"} ! succinate metabolic process

is_a: GO:0015942 {is_inferred="true"} ! formate metabolic process

is_a: GO:0019660 ! glycolytic fermentation

intersection_of: GO:0019660 ! glycolytic fermentation

intersection_of: has_input CHEBI:17234 ! glucose

intersection_of: has_output CHEBI:15740 ! formate

intersection_of: has_output CHEBI:16236 ! ethanol

intersection_of: has_output CHEBI:16810 ! 2-oxoglutarate(2-)

intersection_of: has_output CHEBI:24996 ! lactate

intersection_of: has_output CHEBI:30031 ! succinate(2-)

intersection_of: has_output CHEBI:30089 ! acetate

[Term]

id: GO:0061609

name: fructose-1-phosphate aldolase activity

namespace: molecular_function

def: "Catalysis of the reaction: D-fructose-1-phosphate = dihydroxyacetone phosphate + D-glyceraldehyde." [GOC:dph, GOC:glycolysis, ISBN:0201090910, RHEA:30854]

xref: RHEA:30854

is_a: GO:0016832 ! aldehyde-lyase activity

created_by: dph

creation_date: 2014-03-28T08:54:24Z

[Term]

id: GO:0061610

name: glycerol to glycerone phosphate metabolic process

namespace: biological_process

def: "The chemical reactions and pathways in which glycerol, 1,2,3-propanetriol, is converted to glycerone phosphate." [GOC:dph, ISBN:0201090910]

synonym: "glycerol metabolism to DHAP" EXACT [GOC:dph]

synonym: "glycerol metabolism to dihydroxyacetone phosphate" EXACT [GOC:dph]

synonym: "glycerol metabolism to glycerone phosphate" EXACT [GOC:dph]

synonym: "glycerol to DHAP metabolic process" EXACT [GOC:dph]

synonym: "glycerol to dihydroxyacetone phosphate metabolic process" EXACT [GOC:dph]

is_a: GO:0006071 ! glycerol metabolic process

is_a: GO:0006796 {is_inferred="true"} ! phosphate-containing compound metabolic process

is_a: GO:0019637 {is_inferred="true"} ! organophosphate metabolic process

is_a: GO:0042180 {is_inferred="true"} ! cellular ketone metabolic process

is_a: GO:1901135 {is_inferred="true"} ! carbohydrate derivative metabolic process

intersection_of: GO:0006071 ! glycerol metabolic process

intersection_of: has_input CHEBI:17754 ! glycerol

intersection_of: has_output CHEBI:16108 ! glycerone phosphate

intersection_of: has_part GO:0004367 ! glycerol-3-phosphate dehydrogenase [NAD+] activity

intersection_of: starts_with GO:0004370 ! glycerol kinase activity

created_by: dph

creation_date: 2014-04-03T13:26:21Z

[Term]

id: GO:0061611

name: mannose to fructose-6-phosphate metabolic process

namespace: biological_process

def: "The chemical reactions and pathways in which mannose, the aldohexose manno-hexose, is converted to fructose-6-phosphate." [GOC:dph, ISBN:0201090910, ISBN:0879010479]

synonym: "mannose metabolism to fructose-6-phosphate" EXACT [GOC:dph]

is_a: GO:0006002 {is_inferred="true"} ! fructose 6-phosphate metabolic process

is_a: GO:0006013 ! mannose metabolic process

is_a: GO:0044763 {is_inferred="true"} ! single-organism cellular process

intersection_of: GO:0008152 ! metabolic process

intersection_of: ends_with GO:0004476 ! mannose-6-phosphate isomerase activity

intersection_of: has_input CHEBI:37684 ! mannose

intersection_of: has_output CHEBI:15946 ! keto-D-fructose 6-phosphate

intersection_of: starts_with GO:0019158 ! mannokinase activity

created_by: dph

creation_date: 2014-04-03T13:38:50Z

[Term]

id: GO:0061612

name: galactose to glucose-1-phosphate metabolic process

namespace: biological_process

def: "The chemical reactions and pathways in which galactose, the aldohexose galacto-hexose, is converted to glucose-1-phosphate." [GOC:dph, ISBN:0201090910, ISBN:0879010479]

is_a: GO:0006012 ! galactose metabolic process

is_a: GO:0044763 {is_inferred="true"} ! single-organism cellular process

intersection_of: GO:0006012 ! galactose metabolic process

intersection_of: ends_with GO:0017103 ! UTP:galactose-1-phosphate uridylyltransferase activity

intersection_of: has_input CHEBI:28260 ! galactose

intersection_of: has_intermediate CHEBI:18307 ! UDP-D-galactose

intersection_of: starts_with GO:0004335 ! galactokinase activity

relationship: has_part GO:0003978 ! UDP-glucose 4-epimerase activity

created_by: dph

creation_date: 2014-04-04T08:18:49Z

[Term]

id: GO:0061613

name: glycolytic process from glycerol

namespace: biological_process

def: "The glycolytic process in which glycerol is catabolized to pyruvate generating ATP and NADH." [GOC:dph, ISBN:0201090910]

is_a: GO:0006096 ! glycolytic process

is_a: GO:0006735 {is_inferred="true"} ! NADH regeneration

is_a: GO:0006796 {is_inferred="true"} ! phosphate-containing compound metabolic process

is_a: GO:0019563 {is_inferred="true"} ! glycerol catabolic process

is_a: GO:0019637 {is_inferred="true"} ! organophosphate metabolic process

is_a: GO:0042180 {is_inferred="true"} ! cellular ketone metabolic process

is_a: GO:1901135 {is_inferred="true"} ! carbohydrate derivative metabolic process

intersection_of: GO:0006096 ! glycolytic process

intersection_of: has_input CHEBI:17754 ! glycerol

intersection_of: has_output CHEBI:16908 ! NADH

intersection_of: has_part GO:0004807 ! triose-phosphate isomerase activity

intersection_of: has_participant CHEBI:15846 ! NAD(+)

intersection_of: starts_with GO:0061610 ! glycerol to glycerone phosphate metabolic process

created_by: dph

creation_date: 2014-04-04T08:26:57Z

[Term]

id: GO:0061615

name: glycolytic process through fructose-6-phosphate

namespace: biological_process

def: "The chemical reactions and pathways resulting in the breakdown of a monosaccharide into pyruvate, occurring through a fructose-6-phosphate intermediate, with the concomitant production of ATP and NADH." [GOC:dph, ISBN:0201090910, ISBN:0879010479]

synonym: "glycolysis through fructose-6-phosphate" EXACT [GOC:dph]

xref: MetaCyc:PWY-1042

xref: MetaCyc:PWY-5484

is_a: GO:0006096 ! glycolytic process

is_a: GO:0006735 {is_inferred="true"} ! NADH regeneration

intersection_of: GO:0006096 ! glycolytic process

intersection_of: has_output CHEBI:16908 ! NADH

intersection_of: has_part GO:0003872 ! 6-phosphofructokinase activity

intersection_of: has_part GO:0004332 ! fructose-bisphosphate aldolase activity

intersection_of: has_part GO:0004807 ! triose-phosphate isomerase activity

intersection_of: has_participant CHEBI:15846 ! NAD(+)

created_by: dph

creation_date: 2014-04-08T10:08:49Z

[Term]

id: GO:0061616

name: glycolytic process from fructose through fructose-6-phosphate

namespace: biological_process

def: "The glycolytic process through fructose-6-phosphate in which fructose is catabolized into pyruvate." [GOC:dph, ISBN:0201090910, ISBN:0879010479]

synonym: "glycolysis from fructose through fructose-6-phosphate" EXACT [GOC:dph]

xref: MetaCyc:PWY-5484

is_a: GO:0006001 {is_inferred="true"} ! fructose catabolic process

is_a: GO:0061615 ! glycolytic process through fructose-6-phosphate

intersection_of: GO:0061615 ! glycolytic process through fructose-6-phosphate

intersection_of: has_input CHEBI:28757 ! fructose

intersection_of: starts_with GO:0008865 ! fructokinase activity

created_by: dph

creation_date: 2014-04-08T10:20:37Z

[Term]

id: GO:0061619

name: glycolytic process from mannose through fructose-6-phosphate

namespace: biological_process

def: "The chemical reactions and pathways resulting in the breakdown of mannose into pyruvate, occurring through a fructose-6-phosphate intermediate, with the concomitant production of ATP and NADH." [GOC:dph, ISBN:0201090910, ISBN:0879010479]

is_a: GO:0006002 {is_inferred="true"} ! fructose 6-phosphate metabolic process

is_a: GO:0019309 {is_inferred="true"} ! mannose catabolic process

is_a: GO:0061615 ! glycolytic process through fructose-6-phosphate

intersection_of: GO:0061615 ! glycolytic process through fructose-6-phosphate

intersection_of: has_input CHEBI:37684 ! mannose

intersection_of: starts_with GO:0061611 ! mannose to fructose-6-phosphate metabolic process

created_by: dph

creation_date: 2014-04-14T09:13:13Z

[Term]

id: GO:0061620

name: glycolytic process through glucose-6-phosphate

namespace: biological_process

def: "The chemical reactions and pathways resulting in the breakdown of a carbohydrate into pyruvate, occurring through a glucose-6-phosphate intermediate, with the concomitant production of a small amount of ATP." [GOC:dph, ISBN:0201090910, ISBN:0879010479]

xref: MetaCyc:GLYCOLYSIS

is_a: GO:0061615 ! glycolytic process through fructose-6-phosphate

intersection_of: GO:0061615 ! glycolytic process through fructose-6-phosphate

intersection_of: has_part GO:0004347 ! glucose-6-phosphate isomerase activity

created_by: dph

creation_date: 2014-04-14T09:17:48Z

[Term]

id: GO:0061621

name: canonical glycolysis

namespace: biological_process

def: "The glycolytic process that begins with the conversion of glucose to glucose-6-phosphate by glucokinase activity. Glycolytic processes are the chemical reactions and pathways resulting in the breakdown of a carbohydrate into pyruvate, with the concomitant production of a small amount of ATP." [GOC:dph, ISBN:0201090910, ISBN:0879010479]

xref: MetaCyc:ANAGLYCOLYSIS-PWY

xref: MetaCyc:P341-PWY

xref: MetaCyc:PWY66-40

xref: Reactome:275254 "Glycolysis, Saccharomyces cerevisiae"

xref: Reactome:REACT_115767 "Glycolysis, Gallus gallus"

xref: Reactome:REACT_1383 "Glycolysis, Homo sapiens"

xref: Reactome:REACT_277691 "Glycolysis, Sus scrofa"

xref: Reactome:REACT_284496 "Glycolysis, Gallus gallus"

xref: Reactome:REACT_293115 "Glycolysis, Arabidopsis thaliana"

xref: Reactome:REACT_298380 "Glycolysis, Dictyostelium discoideum"

xref: Reactome:REACT_302664 "Glycolysis, Oryza sativa"

xref: Reactome:REACT_304275 "Glycolysis, Schizosaccharomyces pombe"

xref: Reactome:REACT_313542 "Glycolysis, Canis familiaris"

xref: Reactome:REACT_314687 "Glycolysis, Mus musculus"

xref: Reactome:REACT_319845 "Glycolysis, Drosophila melanogaster"

xref: Reactome:REACT_325720 "Glycolysis, Plasmodium falciparum"

xref: Reactome:REACT_328358 "Glycolysis, Xenopus tropicalis"

xref: Reactome:REACT_333435 "Glycolysis, Bos tarus"

xref: Reactome:REACT_337203 "Glycolysis, Danio rerio"

xref: Reactome:REACT_339865 "Glycolysis, Caenorhabditis elegans"

xref: Reactome:REACT_342181 "Glycolysis, Rattus norvegicus"

xref: Reactome:REACT_351474 "Glycolysis, Taeniopygia guttata"

xref: Wikipedia:Glycolysis

is_a: GO:0006007 {is_inferred="true"} ! glucose catabolic process

is_a: GO:0061620 ! glycolytic process through glucose-6-phosphate

intersection_of: GO:0061620 ! glycolytic process through glucose-6-phosphate

intersection_of: has_input CHEBI:17234 ! glucose

intersection_of: starts_with GO:0004340 ! glucokinase activity

created_by: dph

creation_date: 2014-04-14T09:21:54Z

[Term]

id: GO:0061622

name: glycolytic process through glucose-1-phosphate

namespace: biological_process

def: "The chemical reactions and pathways through a glucose-1-phosphate intermediate that result in the catabolism of a carbohydrate into pyruvate, with the concomitant production of a small amount of ATP." [GOC:dph, ISBN:0201090910]

is_a: GO:0061620 ! glycolytic process through glucose-6-phosphate

intersection_of: GO:0061620 ! glycolytic process through glucose-6-phosphate

intersection_of: has_part GO:0004614 ! phosphoglucomutase activity

created_by: dph

creation_date: 2014-04-28T08:08:01Z

[Term]

id: GO:0061623

name: glycolytic process from galactose

namespace: biological_process

def: "The chemical reactions and pathways resulting in the breakdown of galactose into pyruvate, with the concomitant production of a small amount of ATP." [GOC:dph, ISBN:0201090910]

is_a: GO:0019388 {is_inferred="true"} ! galactose catabolic process

is_a: GO:0061622 ! glycolytic process through glucose-1-phosphate

intersection_of: GO:0061622 ! glycolytic process through glucose-1-phosphate

intersection_of: starts_with GO:0061612 ! galactose to glucose-1-phosphate metabolic process

created_by: dph

creation_date: 2014-04-28T08:13:38Z

[Term]

id: GO:0061624

name: fructose catabolic process to hydroxyacetone phosphate and glyceraldehyde-3-phosphate

namespace: biological_process

def: "The chemical reactions and pathways resulting in the breakdown of fructose that results in the formation of dihydroxyacetone phosphate and glyceraldehyde-3-phosphate." [GOC:dph, ISBN:0201090910]

is_a: GO:0006001 ! fructose catabolic process

is_a: GO:0019682 {is_inferred="true"} ! glyceraldehyde-3-phosphate metabolic process

intersection_of: GO:0009056 ! catabolic process

intersection_of: has_input CHEBI:28757 ! fructose

intersection_of: has_output CHEBI:17138 ! glyceraldehyde 3-phosphate

intersection_of: has_part GO:0050354 ! triokinase activity

intersection_of: has_part GO:0061609 ! fructose-1-phosphate aldolase activity

intersection_of: starts_with GO:0004454 ! ketohexokinase activity

created_by: dph

creation_date: 2014-04-29T12:58:28Z

[Term]

id: GO:0061625

name: glycolytic process through fructose-1-phosphate

namespace: biological_process

def: "The chemical reactions and pathways resulting in the breakdown of fructose into pyruvate through a fructose-1-phosphate intermediate, with the concomitant production of ATP and NADH." [GOC:dph, ISBN:0201090910]

is_a: GO:0006001 {is_inferred="true"} ! fructose catabolic process

is_a: GO:0006096 ! glycolytic process

is_a: GO:0006735 {is_inferred="true"} ! NADH regeneration

is_a: GO:0019682 {is_inferred="true"} ! glyceraldehyde-3-phosphate metabolic process

intersection_of: GO:0006096 ! glycolytic process

intersection_of: has_input CHEBI:28757 ! fructose

intersection_of: has_output CHEBI:16908 ! NADH

intersection_of: has_part GO:0004807 ! triose-phosphate isomerase activity

intersection_of: has_participant CHEBI:15846 ! NAD(+)

intersection_of: starts_with GO:0061624 ! fructose catabolic process to hydroxyacetone phosphate and glyceraldehyde-3-phosphate

created_by: dph

creation_date: 2014-04-29T13:04:10Z

[Term]

id: GO:0061633

name: transport-coupled glycolytic process through glucose-6-phosphate

namespace: biological_process

def: "The chemical reactions and pathways resulting in the breakdown of glucose into pyruvate, in which the glucose is converted to glucose-6-phosphate intermediate coupled to transmembrane transport." [GOC:dph]

is_a: GO:0006007 {is_inferred="true"} ! glucose catabolic process

is_a: GO:0061620 ! glycolytic process through glucose-6-phosphate

intersection_of: GO:0061620 ! glycolytic process through glucose-6-phosphate

intersection_of: has_input CHEBI:17234 ! glucose

intersection_of: starts_with GO:0022855 ! protein-N(PI)-phosphohistidine-glucose phosphotransferase system transporter activity

created_by: dph

creation_date: 2014-06-19T09:12:37Z

[Term]

id: GO:0061688

name: glycolytic process via Entner-Doudoroff Pathway

namespace: biological_process

def: "A glycolytic process in which the glucose is catabolized to pyruvate by first entering the Entner-Doudoroff pathway to yield pyruvate and glyceraldehyde-3-phosphate. The glyceraldehyde-3-phosphate is subsequently converted to pyruvate by the core glycolytic enzymes." [GOC:dph, PMID:9657988]

is_a: GO:0006096 ! glycolytic process

intersection_of: GO:0006096 ! glycolytic process

intersection_of: starts_with GO:0061678 ! Entner-Doudoroff pathway

created_by: dph

creation_date: 2015-03-12T14:39:00Z

[Term]

id: GO:0061704

name: glycolytic process from sucrose

namespace: biological_process

def: "The chemical reactions and pathways resulting in the breakdown of a sucrose into pyruvate, with the concomitant production of a small amount of ATP and the reduction of NAD(P) to NAD(P)H. Glycolysis begins with the metabolism of a carbohydrate to generate products that can enter the pathway and ends with the production of pyruvate. Pyruvate may be converted to acetyl-coenzyme A, ethanol, lactate, or other small molecules." [GOC:dph, GOC:glycolysis, PMID:15012287]

is_a: GO:0005987 {is_inferred="true"} ! sucrose catabolic process

is_a: GO:0006096 ! glycolytic process

intersection_of: GO:0006096 ! glycolytic process

intersection_of: has_input CHEBI:17992 ! sucrose

created_by: dph

creation_date: 2015-06-11T13:26:28Z

[Term]

id: GO:0061705

name: sucrose catabolic process to fructose-6-phosphate through glucose and fructose

namespace: biological_process

def: "The chemical reactions and pathways resulting in the breakdown of sucrose, to yield fructose-6-phosphate through both glucose and fructose intermediates." [GOC:dph, GOC:glycolysis, MetaCyc:PWY-621, PMID:15012287]

is_a: GO:0005987 ! sucrose catabolic process

intersection_of: GO:0009056 ! catabolic process

intersection_of: ends_with GO:0004347 ! glucose-6-phosphate isomerase activity

intersection_of: has_input CHEBI:17992 ! sucrose

intersection_of: has_part GO:0004340 ! glucokinase activity

intersection_of: has_part GO:0008865 ! fructokinase activity

intersection_of: starts_with GO:0004575 ! sucrose alpha-glucosidase activity

created_by: dph

creation_date: 2015-06-11T13:32:05Z

[Term]

id: GO:0061706

name: glycolytic process from sucrose through glucose and fructose

namespace: biological_process

def: "The chemical reactions and pathways resulting in the breakdown of sucrose into pyruvate through both glucose and fructose intermediates, with the concomitant production of a small amount of ATP and the reduction of NAD(P) to NAD(P)H. Glycolysis begins with the metabolism of a carbohydrate to generate products that can enter the pathway and ends with the production of pyruvate. Pyruvate may be converted to acetyl-coenzyme A, ethanol, lactate, or other small molecules." [GOC:dph, GOC:glycolysis, MetaCyc:PWY-1042, PMID:15012287]

is_a: GO:0061620 {is_inferred="true"} ! glycolytic process through glucose-6-phosphate

is_a: GO:0061704 ! glycolytic process from sucrose

intersection_of: GO:0061615 ! glycolytic process through fructose-6-phosphate

intersection_of: starts_with GO:0061705 ! sucrose catabolic process to fructose-6-phosphate through glucose and fructose

created_by: dph

creation_date: 2015-06-11T13:40:32Z

[Term]

id: GO:0093001

name: glycolysis from storage polysaccharide through glucose-1-phosphate

namespace: biological_process

def: "The chemical reactions and pathways resulting in the breakdown of a storage polysaccharide into pyruvate through a glucose-1-phosphate intermediate, with the concomitant production of a small amount of ATP and the reduction of NAD to NADH." [GOC:dph, GOC:glycolysis]

is_a: GO:0000272 {is_inferred="true"} ! polysaccharide catabolic process

is_a: GO:0061622 {is_inferred="true"} ! glycolytic process through glucose-1-phosphate

intersection_of: GO:0061622 ! glycolytic process through glucose-1-phosphate

intersection_of: has_input CHEBI:18154 ! polysaccharide
